# Wall Surfaces

**Published:** 1919-04-19

**Authors:** 


### HOSPITAL CONSTRUCTION: THE NEW ERA.

### VI.—Wall Surfaces.

It is perhaps natural that the articles in this series should arouse some interest among firms engaged in the manufacture of building materials, and that specialists in matters of this kind should write to us on their subjects. We are glad that this should be so; and shall always welcome from them or from professional readers any critical or confirmatory remarks in relation to statements or suggestions made in this set of articles. Hospital construction is a very controversial science. Hospital architects can disagree as heartily as doctors, and the disagreement followed by discussion often leads to the discovery of the only thing that matters —truth.

There lies before us a letter from the Zeta Wood-flooring Company, the patentees of a special type of wood block laid in a special way. In mentioning this firm by name, which we do in acknowledgment of their communication and comments, we are not at all suggesting that their rivals, who are many, do not also produce first-rate goods. In fact, we have personal experience of the work of several other specialist firms in this department of work, which has given perfect satisfaction and has fully confirmed the opinion, expressed in our article on floor surfaces, that a good wood-block floor (*i.e.*, good not only in its theoretical methods of shaping, jointing, and laying, but good also in the quality, seasoning and grain-setting of its blocks) is, if properly cared for and *polished*, rather than washed, the best practical surface on the market. The Zeta speciality is that the blocks are not dowelled or tongued, but rely on a peculiar method of attachment to their bed, and to one another. Each block is provided with a slot, or channel, cut right through its substance at a level below its centre. The blocks are also dovetail-rebated at a level below this slot. It is claimed that they obtain not only that hold on the mastic bed which is common to all blocks that have dovetail (or sloped) grooves at their base, but that the slot receives sufficient overflow of the mastic squeezed up in the act of fixing to unite the blocks to one another. The theory is good and we have no reason to doubt its goodness in practice. One point raised by the Zeta Company in their communication is of interest. Some firms rely for steadiness and homogeneity on dowels or tongues. If these tongues or the dowels are fixed too high in the blocks, there is a chance that under hard wear and tear, the surface of the wood may be worn down actually to the level of the tongues or the dowels. When this occurs the life of the floor is virtually over.

The subject of floor surfaces leads very naturally to that of wall surface; a very puzzling and difficult question. Hospital ward walls are generally either of plaster (cement or lime plaster) or of a glazed material such as glazed bricks, or glazed tiles. The primary object in substituting glazed ware of one sort or the other for the ordinary domestic plaster is cleanliness. It is claimed, and with reason, that a smooth glazed surface will not collect or retain dirt and that consequently, it both combats the harbourage of germs and diminishes the recurrent expense of redecoration. This is a claim which may be overestimated. Every brick is surrounded by joints—so is every tile—and the joint surfaces may themselves become dirt harbourers. It is important, therefore, if either of these materials be adopted, that the joints should be kept as small as possible, as impervious as possible, and above all, as flush with the brick or tile surface as they can be made. It is not enough for a wall to look clean. The very fact that it looks clean when it is not may induce people to leave it without attention when it ought to be cleansed. If one comes to think of it, the unloveliness of dirt is a very important cog in the machinery of nature. Things which "don't show the dirt" are often very guilty things in the moral, as well as the physical world, and certainly it is true that in a hospital all dirt should show so that it may cry aloud for removal. So to return to the glazed brick wall or the tile-covered wall, our meaning is that though the enamelled surfaces both look clean and indeed are clean, by reason of the fact that their smoothness does not admit the lodgment of dirt, it is quite possible for their joints, if uneven, to provide ledges and hollows which, though inconspicuous in the general scheme of decoration, are quite large enough to provide liberal house-room for germs. For this reason, we repeat, it is essential that the joints of all such surfaces, whether brick or tile should be most scrupulously levelled up to the general face of the walling. A word may come in here about the capping moulds on the top of tile dados. It is ridiculous to provide an expensively wrought cove at the junction of dado and floor and then to frustrate this precaution by a flat ledge at the top. If, for any reason, the dado surface is necessarily in advance of the general wall surface above it the capping should be designed so as to form a steep smooth slope on which nothing whatever can find a lodgment.

There is a possible argument against enamelled bricks and tiles which, we venture to think, is generally overlooked. It relates to the occasional prevalence in hospitals, as in our homes, of condensation. In a general way it may be said that the more solidly a building is constructed the more liable it is to this very trying and uncomfortable phenomenon. It is rash for any one who has not studied the question thoroughly and scientifically to offer advice on the complex conditions under which the deposit of air moisture on surfaces can be encouraged or prevented. Perhaps it is enough to point out that even in well-warmed buildings it is sometimes impossible to prevent the walls from

☛ The previous articles appeared on January 25, February 8, March 1, 15 and 29, p. 569.

**The New Era**—(*continued*).

getting cold enough to call the water from the air in certain states of atmosphere, and that the hard, smooth surfaces are the worst offenders.

It is difficult to believe that a damp, dripping wall on a muggy day is wholesome, or that it does not, before it dries off, receive and retain injurious particles of dirt which its surface, when dry, would have rejected. Condensation, of course, is often combated by builders. Steel beams, for example, are in ships and in dye-houses, coated over with powdered cork to prevent the water deposit. But we think that the physicists might help the architects with some advice on the subject. It is difficult in a hospital to carry out the ordinary housekeeper's remedy of keeping all windows tightly closed whenever a warm, damp air follows a frost.

The condensation troubles are not confined to the glazed-tile walls, however. Plastered walls can be nearly as bad, especially if they are painted. This leads us to the subject of the decoration of the plastered wall, and in passing it may be remarked that those who have experimented in the promotion of dew deposit (in connection with "dew-pond" construction) have found that certain colours are much more provocative than others. So that possibly some difficulties in this respect may be relieved by a change in the colour scheme! But to consider, in conclusion, the plastered wall and its decoration.

We are very much inclined to consider that the best colouring material for a plastered surface is distemper. Its cost is less than that of oil paint, it is probably much more sanitary in itself, and it lends itself to easy and frequent renewal. This keeps the wards fresh in appearance, and certainly fresher in fact. We often hear it said, "These walls were so well painted ten years ago that they have never wanted anything but a wash down since." Probably it would have been better for everybody if they had not lasted so well—and an occasional change of colour in the wards, if only a small change, actually gives encouragement to those who work in them.

Choice of colour in a ward is enormously important. Very light colours are not essential, and may be very trying to the eyes. Darker tones, provided they are not relied upon as dirt concealers, are very restful, and all the clean bed-linen and the white clothing of sisters and nurses look well against the contrast. One point is especially worth noting. If the patients are provided with bed-jackets of a uniform colour, the colouring of the walls and of these garments should not fight a continual battle, but should offer a perpetual and pleasant harmony. *Nimium ne crede colori*, said the Roman poet. He spoke amiss. You cannot overrate the importance of colour to the sick and to their attendants.

